# Comorbidities in ADHD children treated with methylphenidate: a database study

**DOI:** 10.1186/1471-244X-13-11

**Published:** 2013-01-07

**Authors:** Angela A Kraut, Ingo Langner, Christina Lindemann, Tobias Banaschewski, Ulrike Petermann, Franz Petermann, Rafael T Mikolajczyk, Edeltraut Garbe

**Affiliations:** 1 Department of Clinical Epidemiology, Leibniz Institute for Prevention Research and Epidemiology – BIPS, Achterstrasse 30, Bremen 28359, Germany; 2 Department of Child and Adolescent Psychiatry, Central Institute of Mental Health, University of Heidelberg, Mannheim, J 5, Mannheim, 68159, Germany; 3 Centre for Psychology and Rehabilitation, University of Bremen, Grazer Strasse 2 & 6, Bremen, 28359, Germany; 4 Department of Epidemiology, Helmholtz Centre for Infection Research, Inhoffenstraße 7, 38124 Braunschweig, Germany; 5 Faculty of Human and Health Sciences, University of Bremen, Bremen, Germany

## Abstract

**Background:**

Methylphenidate (MPH) is the most common drug treatment of attention deficit / hyperactivity disorder (ADHD) in children. Treatment with MPH is contraindicated in the presence of certain psychiatric, cerebro- and cardiovascular conditions. We assessed MPH treatment prevalence and incidence and the frequency of comorbid conditions related to these contraindications in new MPH users compared to a control group without ADHD and ADHD medication.

**Methods:**

We used health care data for the years 2004 to 2006 from the German Pharmacoepidemiological Research Database (GePaRD) which includes about 18% of the German population. MPH treatment prevalence and incidence was assessed based on at least one MPH prescription in the given year. In MPH users, the prevalence of psychiatric and other comorbidities was assessed in the quarter of the first MPH prescription and the three preceding quarters, whereas in controls it was assessed in the earliest four quarters of continuous insurance time starting at 01.01.2004 or the start of insurance if this was later. Differences in the presence of comorbid diagnoses between MPH users and controls were tested by logistic regression.

**Results:**

In 2005, 1.5% of all children and adolescents aged 3 to 17 years (2.3% of males and 0.6% of females) received MPH in Germany. The proportion of children with a record of a psychiatric comorbidity in any of the nine ICD categories of diagnoses was substantially higher in new MPH users (83%) compared to controls (20%). Cerebro- and cardiovascular comorbidities were rare in general. Still, among new MPH users, 2% of males and females had a diagnosis of a pre-existing cardiovascular disorder but only 1.2% of controls.

**Conclusions:**

Besides MPH treatment prevalence we first publish age-specific incidence rates for Germany. A high proportion of children who were started on MPH had a record of a psychiatric comorbidity preceding the first prescription. Cerebro- and cardiovascular conditions were rare in the studied age range, but still higher among children who received MPH than in the control group. Results show that in a substantial subgroup of patients, comorbidities require a thorough weighting of possible risks of MPH medication against the risks of untreated ADHD.

## Background

The stimulant methylphenidate (MPH) is the most common drug treatment for attention-deficit/hyperactivity disorder (ADHD) in children and adolescents [1,2]. MPH is licensed in Germany for the treatment of ADHD in children older than six years of age and for the very rare sleep disorder narcolepsy without any age restriction [3]. In Germany, MPH was launched in 1954 and there was a substantial increase in the volume of prescriptions over the last two decades. Between 1990 and 2009, the number of prescribed daily doses of MPH has multiplied by a factor of 184 [4,5]. While MPH treatment prevalence based on health care data was reported for different age groups, years and countries [2,6-10], treatment incidence was reported less often [11-13].

Drug safety of stimulants has been studied extensively [14-16]. Published case reports of adverse cardiovascular events in persons taking stimulant medication [17], and the well-described effect of these agents to elevate blood pressure and heart rate [16,18,19], have led to public health and regulatory concerns regarding the cerebro- and cardiovascular safety [20]. While cardio- and cerebrovascular conditions are generally rare in childhood, it is well established that psychiatric comorbidity is considerable in children diagnosed with ADHD [21,22].

A safety evaluation of MPH-containing medicines requested by the European Commission and conducted by the European Medicines Agency (EMA) has resulted in a revised list of contraindications which, amongst others, includes pre-existing cerebrovascular, cardiovascular and psychiatric conditions, glaucoma, phaeochromocytoma and hyperthyroidism [23,24]. This safety evaluation has led to a Europe-wide standardisation of the information contained in the Summary of Product Characteristics (SPC) for MPH-containing products, which were implemented in Germany by mid-2009. The investigation of the prevalence of co-morbid conditions newly contraindicated in ADHD children who receive MPH provides an estimation of the magnitude of possibly affected children by the EMA safety evaluation.

The aim of the current study was to assess the prevalence and incidence of MPH prescribing and the extent of pre-existing comorbidity at the time of the first MPH prescription in comparison to a control group without MPH and without ADHD preceding the EMA referral. The specific aims were: 1) to estimate MPH treatment prevalence and incidence in children and adolescents aged 3 to 17 years in Germany 2) to compare the prevalence of psychiatric and other relevant comorbidities in MPH users with a control group.

## Methods

### Data source

This study was based on data from the German Pharmacoepidemiological Research Database (GePaRD) [25]. GePaRD consists of records of four statutory health insurance companies (SHIs) and comprises data of more than 15 million insurance members of all ages (approximately 18% of the German population). The database covers all regions of Germany and includes demographic information and information on hospitalisations, outpatient care and outpatient prescriptions. All diagnoses are coded according to the German Modification of the International Classification of Diseases, 10^th^ Revision (ICD-10-GM). Outpatient diagnoses are accompanied by an indicator for their diagnostic certainty with the specifications ‘certain’, ‘history of’, ‘suspicion of’ and ‘exclusion of’. Outpatient diagnoses can only be allocated to a quarter of the year, since outpatient physician visits are reimbursed quarterly. Statutory health insurance is mandatory in Germany except for persons above a relatively high income threshold who are permitted to choose private health insurance instead. However, also persons above the income threshold can choose to remain in statutory health insurance as it provides free membership for family members without an income of their own. In total, about 90% of the German population is insured in one of several statutory health insurance companies. Preliminary analyses regarding the age and sex distribution, the number of hospital admissions and drug use have shown the database to be adequately representative of information published in official statistics [25]. The current study was based on data for the years 2004 to 2006, since more recent data were not available for all four SHIs at the time of the analysis.

In Germany, the use of health insurance data for scientific research is regulated by the Code of Social Law (SGB X). Approval for the use of the data for this project was granted by all SHIs that contributed data, the Federal Ministry of Health, and the Senate of the Federal State of Bremen. No informed consent was required as the analysis is based on routinely collected, pseudonymised data. The data is not publicly available but other institutions could apply for the data to the respective SHIs.

### Study design and measures

For the inclusion of a child into the study, the following three conditions had to be satisfied: (1) valid information on year of birth and sex; (2) age between 3 and 17 years in the respective year; (3) residence in Germany. Sex- and age-specific MPH treatment prevalence and incidence were calculated for 2005 and 2006. All MPH prescriptions were identified using the anatomic-therapeutic-chemical (ATC) classification code N06BA04.

### Assessment of MPH treatment prevalence

For the assessment of MPH treatment prevalence, all insurees who were insured at least one day in the respective study year were included. The number of insurees for whom at least one MPH prescription was filled in the respective year was divided by all insurees ever insured during that year.

### Assessment of MPH treatment incidence

We included those persons in the analysis who were continuously insured for at least 366 days between 01.01.2004 and 31.12.2006 and did not have an MPH prescription in the first 12 months of their insurance period. Incident MPH users were those who had at least one MPH prescription in 2005 or 2006. We determined MPH treatment incidence as cumulative incidence by dividing the number of incident MPH users by the total number of insured persons stratified by age and sex for the years 2005 and 2006.

### Assessment of comorbidities

For the assessment of differences in comorbidity, we compared comorbid disorders in incident MPH users with those of a control group. In MPH users, we assessed comorbidity in the quarter of the first MPH prescription and the three preceding quarters. As control group we selected insurees who did not fill a prescription for MPH or atomoxetine and who had no diagnosis of hyperkinetic disorders (ICD-10-GM code F90) during the entire study period. Comorbidity in the control group was assessed in the earliest four quarters of continuous insurance time starting at 01.01.2004 or the start of insurance if this was later.

For the analysis of comorbidities we included hospital and outpatient diagnoses, but excluded outpatient diagnoses recorded as ‘exclusion of’, ‘suspicion of’ or ‘history of’. The assessment of psychiatric comorbidity included all ICD-10-GM codes for mental and behavioural disorders apart from the codes F00-F09 which refer to dementia and other organic mental disorders. Psychiatric comorbidities were stratified according to the upper-level groups of the ICD-10 classification scheme (F10-F19, F20-F29, F30-F39, F40-F48, F50-F59, F60-F69, F70-F79, F80-89, F91-F98, and F99). Of these, the frequency of the psychiatric disorders bipolar affective disorder (F31), depression including depressive episode and recurrent depressive disorder (F32 and F33), generalized anxiety disorder (F41.1), conduct disorder (F91) and oppositional defiant disorder (F91.3) was determined individually. Further, comorbid conditions which were listed as contraindications by the European Medicines Agency (EMA) were assessed [23]. A full list of ICD-10-GM codes included for the assessment of comorbidity is available on request.

### Statistical analysis

For the MPH treatment prevalence and incidence estimates 95% confidence intervals (95% CI) were calculated following the method recommended by Newcombe and Altman [26]. To obtain representative prevalence and incidence estimates for the German population, sex- and age-specific estimates were weighted by corresponding population weights for Germany, derived from the German Federal Statistical Office [27]. Logistic regression adjusting for age as categorical variable in one-year age groups was used to calculate p-values for differences in the presence of comorbid diagnoses between users and the control group. P-values of 0.05 or smaller were regarded statistically significant. All statistical analyses were conducted using the statistical program package SAS version 8.2.

## Results

### MPH treatment prevalence and incidence

In total, 2,150,362 insurees aged 3 to 17 years were included for the estimation of MPH treatment prevalence and about the same number of children (N=2,089,877) for the estimation of MPH treatment incidence. The overall treatment prevalence for the 3- to 17-year-old population of Germany was 14.7 per 1,000 insurees in 2005 and 16.9 per 1,000 insurees for 2006 and was about four times higher for males than for females (Table 1).

**Table 1:** Prevalence and incidence of methylphenidate treatment per 1,000 children and adolescents aged 3 to 17 years in 2005 and 2006

	**Year**	**Male (95% CI)**	**Female (95% CI)**	**Total (95% CI)**
**Treatment prevalence**^1^	2005	23.35 (23.05-23.65)	5.50 (5.35-5.65)	14.66 (14.49-14.83)
	2006	26.70 (26.38-27.02)	6.59 (6.43-6.75)	16.90 (16.72-17.09)
**Treatment incidence**^1^	2005	6.65 (6.48-6.81)	1.87 (1.78-1.96)	4.32 (4.23-4.41)
	2006	7.20 (7.03-7.37)	2.16 (2.07-2.26)	4.75 (4.65-4.84)

^1 ^Weighted for the German population aged 3 to 17 years in the respective year, taking age and federal state into account.

**Figure 1:**
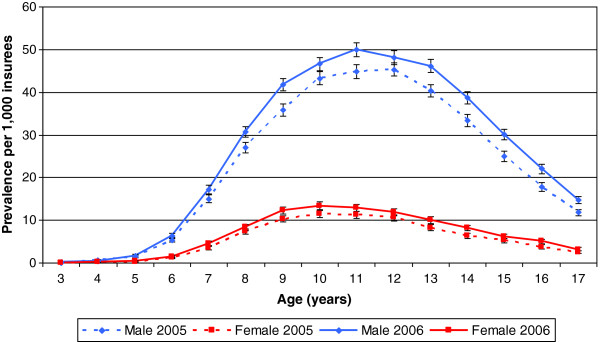
**Prevalence of MPH prescriptions per 1,000 children and adolescents by age and sex in 2005 and 2006. **Note: Indicators represent 95% confidence intervals.

When treatment prevalence was further stratified by age, boys showed a marked increase from the age of 6 with a peak prevalence at age 11, followed by a steep decrease from the age of 13 years. For girls, the population receiving MPH increased from the age of 6 to a maximum at the age of 10 and slightly decreased thereafter. For males, the peak prevalence at age 11 was 44.8 and 49.9 per 1,000 insurees in 2005 and 2006, respectively. For girls, the peak prevalence at age 10 was 11.5 per 1000 insurees in 2005 and 13.4 per 1,000 insurees in 2006. The treatment prevalence was somewhat higher in 2006 than in 2005, which was more marked for males (Figure 1).

**Figure 2:**
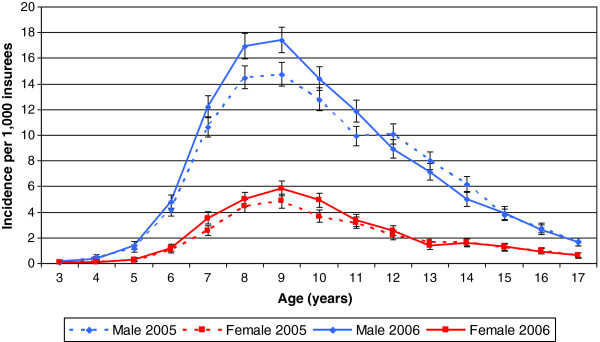
**Incidence of MPH prescriptions per 1,000 children and adolescents by age and sex in 2005 and 2006. **Note: Indicators represent 95% confidence intervals.

The initiation of MPH treatment stratified by age and sex revealed a similar picture. For boys, the incidence of MPH treatment was 6.7 per 1,000 male insurees for 2005 (7.2 per 1,000 insurees for 2006) and 1.9 per 1,000 female insurees in 2005 (2.2 per 1,000 insurees for 2006). The MPH treatment incidence peaked at age 9 for both sexes (Figure 2). Differences between the two years were more marked for boys than for girls.

### Comorbidity

Overall, psychiatric comorbidity was statistically significantly higher in incident users of MPH than in controls for all included diagnostic groups, including the very rare disorders ‘mental and behavioural disorders due to psychoactive substance use’, ‘schizophrenia, schizotypal and delusional disorders’ and ‘unspecified mental disorders’. About half of all incident MPH users had a diagnosis of disorders of psychological development and also about one half had a diagnosis of ‘Behavioral and emotional disorders with onset usually occurring in childhood and adolescence’. ‘Neurotic, stress related and somatoform disorders’ were the third most common psychiatric comorbidity in MPH users, followed by the specific category ‘conduct disorder’. Diagnoses of the other specific psychiatric comorbidities ‘bipolar affective disorder’, ‘depression’, and ‘oppositional defiant disorder’ were present in less than four percent of MPH users and in about one percent or less of controls (Table 2).

**Table 2:** Frequency of psychiatric comorbidities in incident methylphenidate users and controls in 2005 to 2006

	**Males**			**Females**		
**Diagnostic Group (ICD-10-German Modification Codes)**	**Incident users**	**Controls**	**P-value**	**Incident users**	**Controls**	**P-value**
	**(N=13,460)%**^**1**^	**(N=978,790)%**^**1**^		**(N=3,837)%**^**1**^	**(N=1,000,300)%**^**1**^	
**Mental and behavioural disorders (F10-F99)**^2^	**82.52**	**21.53**	**<0.01**	**82.28**	**18.64**	**<0.01**
Mental and behavioural disorders due to psychoactive substance use (F10-F19)	0.81	0.30	<0.01	1.15	0.34	<0.01
Schizophrenia, schizotypal and delusional disorders (F20-F29)	0.35	0.06	<0.01	0.47	0.07	<0.01
Mood [affective] disorders (F30-F39)	3.87	0.48	<0.01	5.40	0.69	<0.01
Bipolar affective disorder (F31)	0.01	0.00^3^	0.03	0.08	0.01	<0.01
Depression (F32, F33)	2.54	0.33	<0.01	3.62	0.55	<0.01
Neurotic, stress-related and somatoform disorders (F40-F48)	19.81	3.58	<0.01	25.70	4.88	<0.01
Generalized anxiety disorder (F41.1)	0.57	0.18	<0.01	1.09	0.23	<0.01
Behavioural syndromes associated with physiological disturbances and physical factors (F50–F59)	5.04	1.18	<0.01	6.18	1.49	<0.01
Disorders of adult personality and behaviour (F60-F69)	10.36	1.11	<0.01	9.59	1.11	<0.01
Mental retardation (F70-F79)	3.80	0.58	<0.01	5.08	0.44	<0.01
Disorders of psychological development (F80-F89)	52.25	13.87	<0.01	48.45	9.90	<0.01
Behavioural and emotional disorders with onset usually occurring in childhood and adolescence^2^ (F91-F98)	55.94	6.03	<0.01	54.29	4.38	<0.01
Conduct disorder (F91)	14.78	1.15	<0.01	11.02	0.81	<0.01
Oppositional defiant disorder (F91.3)	3.65	0.18	<0.01	3.08	0.11	<0.01
Unspecified mental disorder (F99)	1.11	0.20	<0.01	1.59	0.26	<0.01

^1 ^Percentages add up to more than 100% since insurees could have more than one type of comorbidity.

^2 ^Codes for hyperkinetic disorders (F90.x) were excluded from this group.

^3 ^The percentage of male controls with a respective diagnosis was 0.004 (N=41).

The physical comorbidities included in this analysis were quite rare, with most of them present in less than 1% in both, incident users and controls. The most common physical comorbidities were pre-existing cardiovascular disorders, followed by hyperthyroidism and haemodynamically significant heart disease and were statistically significantly more common in incident MPH users compared to controls. Most other cardiovascular comorbidities showed no differences between MPH users and controls. The prevalence of glaucoma was similar in both groups. No diagnoses of cerebral aneurism or pheochromocytoma could be identified in our study (Table 3).

**Table 3:** Selected physical comorbidities in incident methylphenidate users and controls in 2005 to 2006

	**Male**			**Female**		
**Diagnostic Group**	**Incident users**	**Controls**	**P-value**	**Incident users**	**Controls**	**P-value**
	**(N=13,460)%**^**1**^	**(N=978,790)%**^**1**^		**(N=3,837)%**^**1**^	**(N=1,000,300)%**^**1**^	
Pre-existing cardiovascular disorders	2.00	1.24	<0.01	2.06	1.18	<0.01
Angina pectoris	0.13	0.19	0.34	0.16	0.18	0.90
Arterial occlusive disease	0.01	0.02	0.98	0.08	0.03	0.04
Potentially life threatening arrhythmias	0.51	0.17	<0.01	0.57	0.14	<0.01
Cardiomyopathy	0.10	0.05	0.02	0.03	0.05	0.57
Severe congenital heart disease	1.23	0.78	<0.01	1.20	0.75	<0.01
Heart failure	0.09	0.05	0.02	0.03	0.05	0.69
Myocardial infarction	0.01	0.01	0.64	0.03	0.02	0.43
Severe hypertension	0.07	0.05	0.34	0.05	0.05	0.82
Pre-existing cerebrovascular disorders	0.19	0.11	<0.01	0.13	0.10	0.36
Stroke	0.10	0.05	0.02	0.10	0.04	0.02
Glaucoma	0.39	0.33	0.34	0.34	0.35	0.96
Hyperthyroidism	1.22	0.15	<0.01	0.89	0.27	<0.01

^1 ^Percentages could add up to more than 100% since insurees could have more than one type of comorbidity.

## Discussion

With this study, we complement previous studies based on health care data and provide prevalence and incidence of MPH treatment in children and adolescents in Germany using a large health care database. Our study showed that the prevalence of psychiatric comorbidities is considerably higher in MPH users compared to controls, whereas the differences for physical comorbidities were less pronounced.

### MPH treatment prevalence

For Germany, MPH treatment prevalence was reported in two regional [2,8] and one national study [6], each based on data from a single health insurance company.

Our findings which are based on data from four health insurance companies covering all parts of Germany are in accordance with those of the national study [6], which reported a treatment prevalence of 1.7% overall and of 2.7% for males and of 0.7% for females in children aged 0 to 17 years in 2006. Another study based on data from the Federal State Mecklenburg-West Pomerania reported a total MPH treatment prevalence of 1.4% in 0- to 15-year-old children in 2001 [8]. A lower MPH treatment prevalence of 1.1% was also reported from Hesse in 2007, with an estimate of 1.7% for boys and 0.4% for girls under the age of 18 [2]. These lower prevalence estimates in Mecklenburg-West Pomerania and Hesse may indicate regional variations in MPH treatment in Germany [8]. In our study, treatment prevalence increased steadily from age 6 to age 11, which was also observed in the study from Hesse [2]. Males had a four times higher treatment prevalence than females which equates to their about fourfold higher prevalence of ADHD diagnosis [28].

### MPH treatment incidence

Treatment incidence overall was 4.75 per 1000 children and adolescents in 2006 and was 3.3-fold higher in male than in female children. No comparable estimates for MPH treatment incidence have been reported for Germany. A nationwide Icelandic study based on prescription data of children aged 0 to 17 years found an incidence of 5.4 per 1,000 children for stimulants and atomoxetine combined for the year 2006 which was 2.5 times higher in boys than in girls [10]. A study from the United States (US) based on Medicaid data reported that 8.9 per 1,000 children and youths below the age of twenty were newly started on any ADHD drug in 2003 to 2004 [13]. This might indicate a different prescribing behaviour in the US. Another study from the Netherlands which was based on a sample of pharmacy medication histories reported an incidence of ADHD drug treatment of 3.10 per 1,000 inhabitants up to the age of 44 years for 2006 [12]. Since this estimate also included adults, it is not surprising that it is lower than our estimate for children only. In our study the MPH treatment incidence increased markedly from the age of 6, the typical age of starting school in Germany, up to the age of 9 years. Similarly, the study from the Netherlands [12] found the MPH treatment incidence to be highest in the 6- to 11-year-old population, compared to the age groups below 6 years of age and 12 to 17 years. No age-specific incidence rates were reported for Germany prior to our study.

### Psychiatric comorbidities

According to the EMA, the presence of a diagnosis or history of severe depression, anorexia nervosa/anorexic disorders, suicidal tendencies, psychotic symptoms, severe mood disorders, mania, schizophrenia, psychopathic/borderline personality disorder, and severe episodic (Type I) bipolar affective disorder are contraindications for the use of MPH [23]. Overall, all investigated psychiatric comorbidities were with 82% very common in children with MPH prescriptions and their overall frequency did not differ between boys and girls. The frequent co-occurrence of ADHD with psychiatric conditions is well known [21,29-31] and was shown in both, epidemiologic [32,33] and clinical studies [21,34-36]. Psychiatric conditions are so frequent and interwoven with ADHD symptoms that the introduction of new ADHD subtypes based on patterns of psychiatric comorbidity has been proposed [22,37]. A Swedish community-based study of 7-year-old school children found a similar psychiatric comorbidity prevalence (87%) for children who, according to a clinical examination and parent and teacher interviews, fulfilled the criteria of the Diagnostic and Statistical Manual of Mental Disorders (DSM-III-R) for ADHD [33].

Generally, studies reporting psychiatric comorbidity in children with ADHD are mainly based on clinical examinations of small patient samples [29,38]. In these studies, children are actively screened for psychiatric comorbid conditions which results in a very high proportion of ADHD children with these comorbidities. Due to these methodological differences, the published estimates are not comparable to ours. An interesting point is, that ADHD diagnoses in our study were defined by ICD-10-GM criteria, which in contrast to the DSM classification demand that the diagnosis ADHD should not be given in the presence of mood disorders, anxiety disorders, schizophrenia or pervasive developmental disorders.

Conduct disorder was the most prevalent specific comorbidity in our study. This accords with a European clinical guideline on ADHD which states that oppositional defiant disorder (ODD) and conduct disorder (CD) are very common in ADHD and could rather be seen as a complication of ADHD than a comorbidity [39]. For Germany, the Attention-deficit/hyperactivity Disorder Observational Research in Europe (ADORE) study has found ODD and/or CD in 46% (n=197) of 6- to 18-year-old patients with ADHD symptoms who had not formally been diagnosed with ADHD before study inclusion [40,41]. The German part of this study included 434 children with ADHD symptoms and was based on standardised physician and parent reported measures in all children which may explain the higher prevalence found in the ADORE study compared to our results. In another small population-based sample of 7-year-old children as many as 60% of those with ADHD had a comorbid diagnosis of ODD [33]. A detailed discussion of the association of conduct disorder with ADHD can be found in a review by Biederman and colleagues, which reports that ODD/CD have been found in 30% to 50% of children with ADHD in clinical and in epidemiological studies [21].

A prospective study showed that children with ADHD are at a 4.3-fold higher risk to develop major depression or dysthymia (according to DSM-IV criteria) through the age of 18 compared to children without ADHD [42]. Another prospective study including adolescent and young adult females with ADHD also using DSM-IV criteria found a 2.5 times higher risk of major depression in the presence of ADHD compared to female controls without ADHD [29]. Due to non-specific coding of depression in the database (F32.9), we could not investigate major depression in children with and without MPH use, however, depression overall was significantly more frequent in male and female children receiving MPH prescriptions than in the respective controls.

Clinical and epidemiological studies have reported that ADHD and anxiety disorders occur together in 8% to 30% of children [31]. A recent review stated that the co-occurrence of anxiety disorders with ADHD averages 25% in these studies [43]. The ADORE study found anxiety and/or depression in 14% (n=57) of German study participants [41]. Anxiety disorders in a wider sense also include obsessive compulsive disorders and a variety of other forms of anxiety disorders. In our study, only generalized anxiety disorders were analysed and occurred rarely, but, as all considered psychiatric comorbidities was significantly more frequent in MPH users compared to controls.

We found only a small number of children and adolescents with a diagnosis of bipolar affective disorder in our study, but still there were statistically significant differences between MPH users and controls. In the literature, bipolar disorder was reported in up to 22% of children with ADHD [43]. The difference can be related to the fact that ICD-10-GM criteria demand that the diagnosis ADHD should not be given in the presence of mood disorders, anxiety disorders, schizophrenia or pervasive developmental disorders.

### Cardiovascular and other comorbidities

A review of adverse drug reaction reports in the US and Europe has led to concerns that MPH treatment may be associated with rare cardiovascular complications [17]. The EMA contraindicated the use of MPH in patients with the following cardiovascular conditions: pre-existing cardiovascular disorders including heart failure, arterial occlusive disease, angina, haemodynamically significant congenital heart disease, cardiomyopathies, myocardial infarction, potentially life-threatening arrhythmias and channelopathies [23]. Meanwhile, two database studies investigating the cardiovascular safety of ADHD drugs have been published which did not show an increased risk for serious cardiovascular outcomes [44,45]. Further contraindications resulting from the EMA safety evaluation are cerebrovascular disorders, phaeochromocytoma, hyperthyroidism or thyrotoxicosis, and glaucoma. A thorough pre-treatment and ongoing screening of the psychiatric and cardiovascular status of the patient is considered necessary by the EMA.

Even though cardiovascular conditions are quite rare in children in general, we found higher prevalence estimates for pre-existing cardiovascular disorders in children who were started on MPH treatment compared to controls, especially for congenital heart diseases and life-threatening arrhythmias. There is some evidence for ADHD being more prevalent in children with heart disease than in the general paediatric population consistent with our findings [46]. A possible explanation for this association is that chronic or intermittent hypoxia has an adverse impact on development, behaviour and academic achievement [47].

Our prevalence estimates of congenital heart disease in controls were somewhat higher than those from a Canadian population-based database study which reported a prevalence of severe congenital heart disease of 0.02 per 100 children and adolescents under the age of 18 years in 2000 [48]. Since no specific estimates for the prevalence of the investigated cardiovascular and other comorbidities in children are published for Germany, it was not possible to compare our estimates of the prevalence of these disorders in the control group with national estimates.

ADHD has been associated with a generalized resistance to thyroid hormone, but this could not be confirmed in clinical studies [49,50]. It is not clear why hyperthyroidism was more common in MPH users in our study. A possible explanation could be given by more frequently performed diagnostic investigations for hyperthyroidism in children with ADHD and, as a consequence, a more frequent detection of clinically non-severe cases.

### Strengths and limitations

The main strengths of this analysis are the large size and the population-based nature of the database. GePaRD consists of records of four statutory health insurance companies and was shown to be representative for Germany regarding the sex and age distribution, hospital admissions and drug prescriptions [25,51]. To compensate for differences in the fraction of persons included in the database across federal states, we weighted the derived estimates with population weights obtained from the Federal Statistical Office [27]. In Germany, MPH belongs to the controlled substances and prescribing is regulated by law. Thus, MPH prescriptions are considered to have a high validity and are easily identifiable in GePaRD.

Our study focussed on description of pre-existing comorbidities among children receiving MPH to investigate the impact of new contraindications implemented by the EMA. MPH is mainly used for the treatment of ADHD and the only other indication it is licensed for in Europe is the very rare condition narcolepsy. Since under-coding of ADHD diagnosis in routine data may occur, we equated children who received MPH treatment with children with ADHD. This might have lead to some misclassification. Among children with ADHD diagnoses, the presence of certain psychiatric comorbidities decreased the probability of drug prescriptions in two US-based studies using Medicaid data [52,53]. This might indicate that comorbidity is less prevalent in MPH-treated patients than in ADHD patients overall, however, we do not know whether these results are transferable to Germany. The higher psychiatric comorbidity in Germany found in the ADORE study, which was based on an investigation of children with ADHD symptoms overall and not only of those treated with MPH, could indicate that this may also apply to Germany.

Due to diagnostic coding by ICD-10-GM codes in the database, we could only assess diagnoses which are coded in this system. Some contraindications for MPH, e.g. potentially life-threatening channelopathies, could not be assessed as they do not have separate codes in the ICD-10 coding system. Also, ICD-10 codes do not give any indication of the severity of the disease. Some psychiatric diagnoses may be under coded as e.g. ODD, explaining the lower prevalence found in our study than in other German studies.

ADHD children are likely under closer surveillance than control children and may additionally be screened for organic causes of their symptoms. For this reason, some bias regarding the detection of comorbidity in ADHD children is possible. Additional analyses (i) of all newly diagnosed ADHD patients and (ii) of all newly diagnosed ADHD patients who started treatment with MPH within 365 days of their ADHD diagnosis in the database showed only slight differences in the prevalence of comorbidities compared to the MPH users in this study. These additional analyses were conducted in children within the same age range as in this study, thereby ruling out possible concerns regarding detection bias in ADHD children as far as possible. Only hyperthyroidism was substantially higher in ADHD children in this study compared to newly diagnosed ADHD children. This might be in fact the result of detection bias as discussed earlier.

Finally, at the time the analysis was conducted, only data for the years 2004 to 2006 were available. For that reason, we were not able to ascertain the impact of the contraindications introduced by the EMA in the beginning of 2009 on prescribing behaviour. Our study provides insight in how many children might be affected by psychiatric comorbidity in general and by these newly agreed contraindications in particular. MPH treatment of ADHD in children with contraindications might outweigh the risks of not treating ADHD in many cases [18,54].

## Conclusions

MPH treatment prevalence was in accordance with the published estimate of a national study, while results from smaller regional studies may indicate regional variations in prescribing behaviour. In this study we reported age-specific treatment incidence rates for the first time. Children starting MPH treatment had a high prevalence of pre-existing psychiatric comorbidities which may affect MPH prescribing. Cardiovascular and other comorbidities were generally rare, but require attention as they were more common among those who received MPH than in the control group.

## Competing interests

AAK received funding from Sanofi Pasteur MSD for the annual conference of the German Society of Epidemiology in 2010. The present work is unrelated to the funding mentioned. TB served in an advisory or consultancy role for Bristol Myers-Sqibb, Develco Pharma, Lilly, Medice, Novartis, Shire, Viforpharma; YES-Pharma. He received conference attendance support and conference support or received speaker’s fee by Lilly, Janssen McNeil, Medice, Novartis and Shire. He is/has been involved in clinical trials conducted by Lilly and Shire. The present work is unrelated to the above grants and relationships. RTM received research funding from Sanofi Pasteur MSD and Bayer-Pharma. The mentioned funding is unrelated to the present work. EG is running a department that occasionally performs studies for pharmaceutical industries with the full freedom to publish. The companies include Mundipharma, Bayer-Pharma, Stada, Sanofi-Aventis, Sanofi-Pasteur, Novartis, Celgene and GSK. In the past, EG has been consultant to Bayer-Schering, Nycomed, Teva and Novartis. The present work is unrelated to the stated relationships. IL, CL, UP and FP declare no competing interests.

## Authors’ contributions

AAK, CL, TB, RTM, and EG were involved in the study conception and study design, and all authors in the interpretation of the results. AAK and IL were involved in drafting the manuscript; IL conceptualised and performed the statistical analysis. All authors have critically revised and approved the final manuscript.

## Pre-publication history

The pre-publication history for this paper can be accessed here:

http://www.biomedcentral.com/1471-244X/13/11/prepub
